# Single-Cell Transcriptomic Profiling Reveals Dual Antitumor and Adaptive Resistance Mechanisms of a Novel HSP90 Inhibitor, SP11, in T-Cell Acute Lymphoblastic Leukemic Cells and DLA Mouse Model

**DOI:** 10.3390/ijms27125321

**Published:** 2026-06-12

**Authors:** Shahana M V, Anjitha R, Bibha Choudhary

**Affiliations:** 1 Center for Doctoral Studies, Manipal Academy of Higher Education, Manipal 576104, India; azadshahana56@gmail.com (S.M.V.);; 2 Institute of Bioinformatics and Applied Biotechnology, Electronics City, Phase I, Bengaluru 560100, India

**Keywords:** HSP90, SP11, ScRNA, leukemia, lymphoma, MYC, BCL2

## Abstract

Heat shock protein 90 (HSP90) is a molecular chaperone essential for maintaining the stability of many oncogenic client proteins. Although several HSP90 inhibitors (HSP90i) have entered clinical trials, their use has been limited by toxicity and resistance, underscoring the need for improved therapeutic strategies. In this study, we assessed the therapeutic potential of a new HSP90i, SP11, in T-cell acute lymphoblastic leukemia (T-ALL) in vitro and in the DLA mouse model in vivo, using single-cell transcriptomic profiling. Single-cell RNA sequencing showed that SP11 treatment reduces key oncogenic drivers, including MYC, BCL2, and stemness-related genes, consistent with impaired leukemic survival programs. In the DLA mouse model, SP11-mediated HSP90 inhibition was associated with alterations in the tumor microenvironment, including increased immune cell representation and enrichment of cytokine- and antigen-presentation-related transcriptional pathways. Despite these antitumor effects, a distinct subpopulation of cells continued to express or re-express MYC and BCL2, suggesting the development of early adaptive resistance. Consistent with these findings, an SP11-resistant MOLT4 cell line maintained high levels of MYC and BCL2 at both the transcript and protein levels, maintained CD44 expression, and exhibited altered inflammatory cytokine signaling. Functional studies confirmed that pharmacological inhibition of BCL2 notably increased SP11 sensitivity, supporting a rational combination strategy. Collectively, our results show that SP11 may exert both tumor-intrinsic and immune-modulating effects and reveal transcriptionally defined adaptive cellular states linked to resistance. This study provides mechanistic in sights into responses to HSP90 inhibition and supports combination approaches for improving therapeutic outcomes in T-ALL.

## 1. Introduction

T-cell acute lymphoblastic leukemia (T-ALL) is a highly aggressive blood cancer characterized by the uncontrolled growth of immature T-cell precursors. It accounts for about 15% of pediatric and 25% of adult cases of acute lymphoblastic leukemia (ALL) and is often associated with high white blood cell counts at diagnosis [1,2]. Despite major advances in multi-agent chemotherapy treatments, many patients relapse or develop resistant disease, highlighting the urgent need for more effective and targeted therapies. A key clinical challenge in T-ALL is the development of drug resistance, which arises through mechanisms including upregulation of anti-apoptotic pathways, metabolic changes, and activation of compensatory signaling networks [3,4]. Additionally, the survival of leukemia stem cells (LSCs) with self-renewal ability contributes to disease recurrence and therapeutic failure [5].

Cancer stem cells (CSCs), first identified in 1990, are a small but functionally vital subgroup of tumor cells capable of self-renewal, differentiation, and tumor promotion. These cells contribute to intratumoral heterogeneity and exhibit increased resistance to conventional therapies, making them central to tumor progression, metastasis, and relapse [6,7]. A key regulator of CSC maintenance is Heat Shock Protein 90 (HSP90), a molecular chaperone that stabilizes many client proteins involved in cancer-promoting signaling pathways [8]. These client proteins regulate proliferation, survival, and stemness, and their proper function depends on HSP90-assisted folding and stabilization. Therefore, pharmacological inhibition of HSP90 triggers the degradation of several oncogenic drivers simultaneously, disrupting tumor-promoting networks and impairing CSC survival.

SP11 is an innovative HSP90 inhibitor from the coumarin-imidazothiadiazole class, with a unique structure distinct from traditional geldanamycin-based inhibitors. Studies have shown that SP11 triggers intrinsic apoptosis and markedly reduces tumor burden in Dalton’s Lymphoma Ascites (DLA) models [9,10]. These results suggest it has strong potential as a therapeutic agent with enhanced effectiveness and possibly fewer side effects. Nonetheless, as with other HSP90 inhibitors, the development of resistance remains a significant challenge that must be addressed for sustained treatment success.

Recent studies have shown that MYC and BCL2 are key mediators of resistance to HSP90 inhibition in aggressive lymphoid cancers. MYC, a major oncogenic transcription factor and a direct client of HSP90, controls gene expression programs that support cell growth, metabolic reprogramming, and survival. Likewise, BCL2 is a major anti-apoptotic regulator that shields cells from programmed death. The simultaneous activation of MYC and BCL2 creates a robust survival network that drives therapy resistance and poor clinical outcomes [11,12,13]. Although HSP90 inhibition can destabilize these proteins at the post-transcriptional level, tumor cells may bypass this by increasing transcription of MYC- and BCL2-related pathways. Supporting this idea, our earlier analysis of the CLUE database showed consistent activation of MYC and BCL2 signaling pathways across multiple cancer cell lines treated with HSP90 inhibitors [14].

Despite these insights, the cellular heterogeneity and dynamic transcriptional changes that underlie resistance to HSP90 inhibition remain poorly understood, especially in T-ALL. Traditional bulk transcriptomic methods are limited in their ability to detect rare but clinically important subpopulations, such as adaptive/drug-tolerant cells and residual stem-like groups. In this context, single-cell RNA sequencing (scRNA-seq) offers a powerful tool to explore tumor heterogeneity, track changes in cellular states, and identify early adaptive resistance mechanisms in high detail.

In this study, we used single-cell transcriptomic profiling to examine the effects of SP11 in both in vitro (MOLT4) and in vivo (DLA) models of T-ALL. Through clustering, trajectory analysis, and functional validation, we aimed to (i) understand how SP11 influences oncogenic and stemness-related pathways, (ii) explore treatment-induced changes in the tumor microenvironment, and (iii) identify transcriptionally distinct subpopulations linked to adaptive resistance. Our results offer mechanistic insights into the tumor-intrinsic and microenvironmental effects of SP11 and lay the groundwork for developing rational combination strategies to overcome resistance in T-ALL.

## 2. Results

### 2.1. Single-Cell Transcriptomic Profiling Reveals Cellular Heterogeneity Following SP11 Treatment

We generated single-cell RNA-seq profiles of MOLT4 (T-ALL) and DLA (Lymphoma) cells to study cellular diversity and transcriptional responses following treatment with SP11 (an HSP90 inhibitor). Initially, we captured 1076 control and 864 SP11-treated MOLT4 cells, along with 965 control and 1103 SP11-treated DLA cells. Cells with fewer than 200 detected genes or more than 20% mitochondrial gene expression in MOLT4 (or more than 10% in DLA) were removed to ensure data quality. After filtering, we retained 943 control and 850 SP11-treated MOLT4 cells, and 915 control and 1081 SP11-treated DLA cells for analysis. These datasets provided sufficient detail to examine both overall transcriptional changes and specific cellular differences caused by SP11 at the single-cell level.

### 2.2. SP11 Induces Broad Suppression of Oncogenic and Survival-Associated Programs

**Figure 1:**
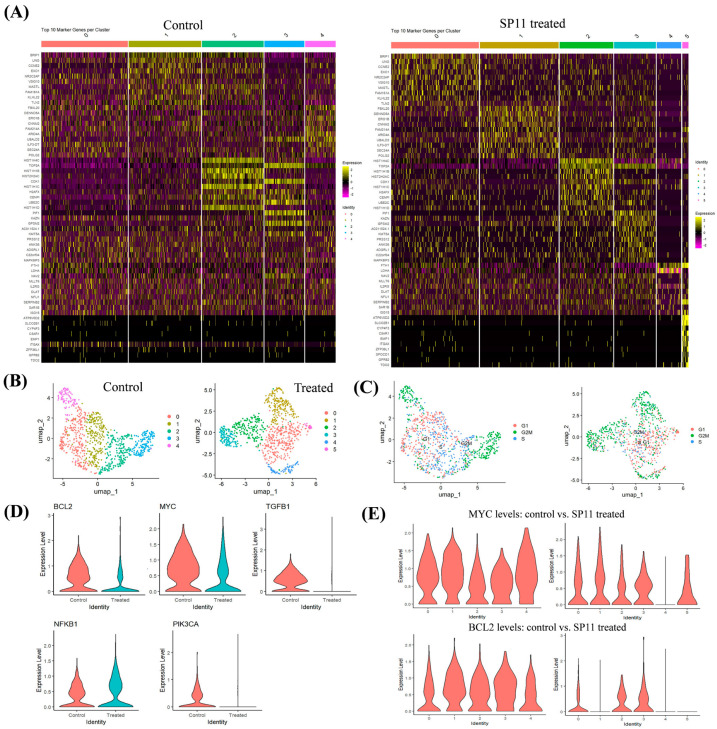
SP11 treatment alters gene expression, cellular clustering, and cell cycle dynamics in MOLT4 cells (**A**) Heatmap showing the top 10 marker genes for each cluster in control (**left**) and SP11-treated (**right**). SP11 treatment significantly altered the transcriptional profile of MOLT4 cells. (**B**) UMAP plots illustrating the distribution of transcriptionally distinct clusters in control (**left**) and treated (**right**). (**C**) Cell cycle phase distribution in control and treated cells. (**D**) Violin plot comparing the expression levels of key regulators (*BCL2*, *MYC*, *TGFB1*, *NFKB1*, *PIK3CA*) between control and treated groups. (**E**) Cluster-wise expression levels of *MYC* and *BCL2* in control versus SP11-treated cells.

Unsupervised clustering of MOLT4 cells identified five transcriptionally distinct clusters in the control group and six clusters in the SP11-treated group, suggesting that treatment alters both cellular composition and transcriptional states (Figure 1A,B). The appearance of an additional cluster in treated cells indicates the induction of new transcriptional states, potentially reflecting adaptive or stress-related phenotypes. To assess whether SP11 caused changes in the cell cycle, we performed cell cycle scoring using Seurat’s CellCycleScoring function. This analysis revealed a decrease in G1- and S-phase cells and a corresponding increase in G2/M-phase cells (Figure 1C,D), indicating cell cycle disruption after treatment. At the overall expression level, SP11 treatment led to significant downregulation of *MYC*, *BCL2*, *TGFB1*, *NFKB1*, and *PIK3CA* (Figure 1D). These genes are key regulators of proliferation, survival, inflammation, and metabolic signaling. Their coordinated suppression provides mechanistic evidence that SP11 effectively inhibits core oncogenic pathways, consistent with its HSP90-inhibitory activity. Consistent with these findings, reduction in *MYC* expression was observed across all clusters and was completely absent in cluster 4, while *BCL2* expression decreased in three clusters and was undetectable in cluster 5 (Figure 1E). These results underscore significant intratumoral heterogeneity in transcriptional responses to SP11 treatment.

### 2.3. Single-Cell Analysis Reveals Heterogeneous Drug Responses and the Emergence of Drug-Tolerant Subpopulations

The emergence of a distinct fifth cluster, observed exclusively in the SP11-treated condition, is a significant finding that indicates the induction of a treatment-associated transcriptional state. This cluster likely represents a drug-tolerant (adaptive) subpopulation that develops under therapeutic pressure. Cluster 5 was characterized by increased expression of *TDO2*, *ZFP36L1*, *ITGAX*, *EMP1*, *FTH1*, *SERPINB2*, *ISG15*, *ATP6V0D2*, *SLCO2B1*, *CYP4F3*, *C5AR1*, *SPOCD1*, and *GPR82* (Figure 1A). This transcriptional profile suggests a treatment-adapted, immunomodulatory, and survival-enhancing phenotype. Notably, *TDO2* indicates activation of the kynurenine-mediated immunosuppressive pathway [15], while *ZFP36L1* has been linked to imatinib resistance [16]. Immune-related genes, including *ITGAX* [17], ISG15 [18,19], and *C5AR1* [20], further support altered immune signaling. Additionally, genes associated with survival and drug response, such as *EMP1* [21], *FTH1* [22], *SERPINB2* [23], *ATP6V0D2* [24], SLCO2B1, and CYP4F3 [25], were strongly expressed. The presence of *SPOCD1* [26] and the leukemia-associated GPCR *GPR82* [27] further supports the existence of a distinct adaptive state. Overall, these findings suggest that Cluster 5 represents a drug-tolerant (adaptive), transcriptionally reprogrammed subpopulation with features of immune modulation and survival adaptation.

### 2.4. Stemness-Associated Genes Are Downregulated Following SP11 Treatment

**Figure 2:**
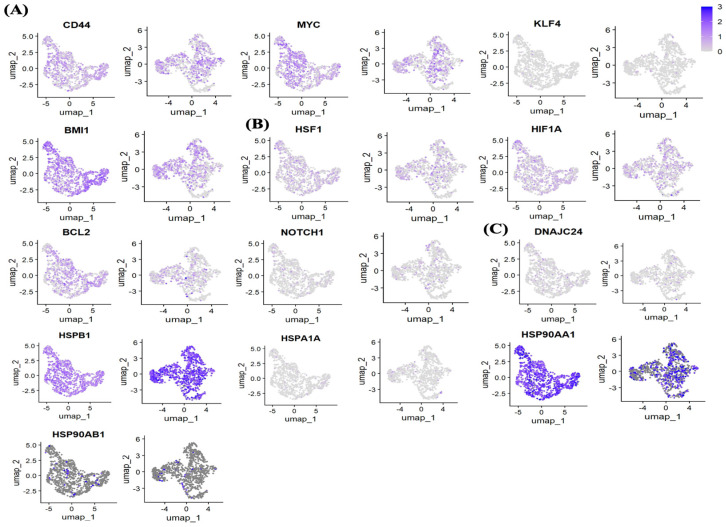
Single-cell RNA-seq cluster analysis of control versus SP11-treated MOLT-4 cells. The UMAP cluster map compares gene expression across treatment conditions. (**A**) Stemness markers—*CD44*, *MYC*, *KLF4*, *BMI1* (**B**) *HSP90* client genes—*HSF1*, *HIF1A*, *BCL2* and *NOTCH1*. (**C**) Other HSPs—*DNAJC24*, *HSPB1*, *HSPA1A*, *HSP90AA1*, *HSP90AB1*.

To assess whether SP11 influences stemness-related programs, we examined the expression of key stemness markers across clusters. SP11 treatment significantly decreased *CD44*, *KLF4*, *MYC*, and *BMI1*, which are vital regulators of self-renewal and tumorigenicity (Figure 2A). This decline indicates impaired stem-like features and reduced tumor-propagating potential after treatment. We also looked at the expression of HSP90 client proteins (*HSF1, HIF1A, BCL2, NOTCH1*) and other heat shock proteins (*DNAJC24*, *HSPB1*, *HSPA1A*, *HSP90AA1*, *HSP90AB1*) to evaluate stress response activation. Most stress-related genes were downregulated following SP11 treatment, supporting effective inhibition of HSP90-mediated signaling pathways. Interestingly, HSPB1 levels were higher, suggesting a possible compensatory stress response that might help develop resistance.

### 2.5. Trajectory Analysis Reveals Cellular Reprogramming and Resistance-Associated States in MOLT4 Cells

**Figure 3:**
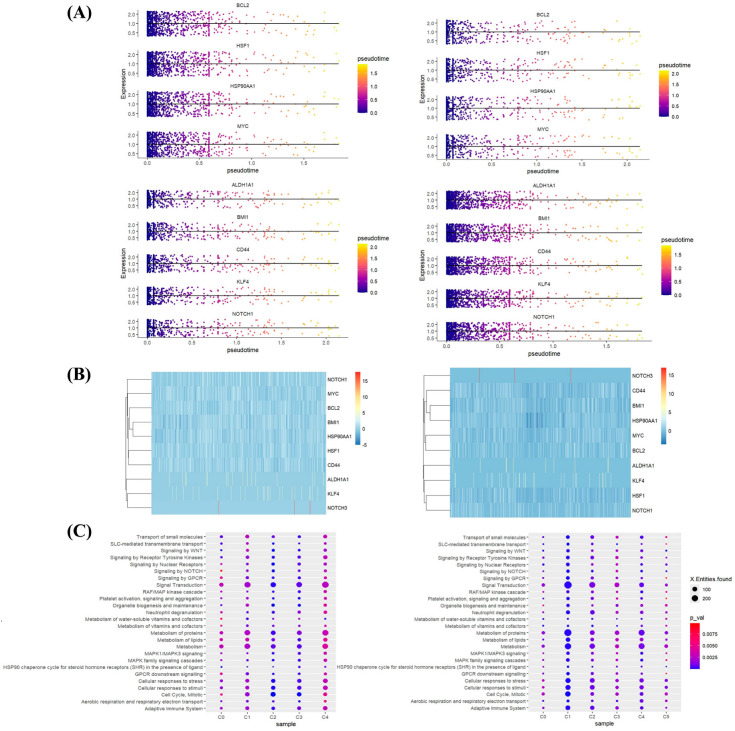
(**A**) Pseudotime trajectory plots showing the expression dynamics of key oncogenic and stemness-related genes (*BCL2*, *HSF1*, *HSP90AA1*, *MYC*, *ALDH1A1*, *BMI1*, *CD44*, *KLF4*, *NOTCH1*, *NOTCH3*) in control (**left**) and SP11-treated (**right**) MOLT4 cells. In controls, these genes show progressive upregulation along pseudotime, while SP11-treated cells display suppressed or delayed expression trajectories, indicating impaired self-renewal and survival. (**B**) Trajectory heatmaps showing the dynamic expression of key oncogenic and stemness-associated genes along pseudotime in control (**left**) and SP11-treated cells (**right**). Each row represents a gene, and color intensity reflects expression levels across the pseudotime trajectory from left (early state) to right (late state). Compared with control cells, SP11-treated cells exhibited lower expression of *MYC*, *BCL2*, *BMI1*, and *NOTCH1* across the trajectory, suggesting modest changes in stemness- and proliferation-associated transcriptional patterns during developmental progression. (**C**) Dot plots representing pathway enrichment analysis across clusters (C0–C4) in control (**left**) and SP11-treated (**right**) samples. Pathways related to metabolism, *GPCR* signaling, receptor tyrosine kinases, Signal transduction, cell cycle, and immune responses are highly enriched in control clusters. In contrast, SP11-treated cells exhibited relatively lower enrichment of several oncogenic signatures. The size of each dot corresponds to the number of entities identified within the respective pathway.

To further explore treatment-induced cellular transitions, trajectory analysis was performed to reconstruct dynamic transcriptional changes and identify potential resistance-related states. Gene-wise expression scatter plots showed distinct differential expression patterns across key regulatory and functional genes, indicating notable transcriptional reprogramming following SP11 treatment (Figure 3A). These differences were further supported by hierarchical clustering of selected differentially expressed genes (*MYC*, *BCL2*, *NOTCH1*, *NOTCH3*, *BMI1*, *CD44*, *HSF1*, *KLF4*, *HSP90AA1*, and *ALDH1A1*), which revealed clear separation of samples by condition, underscoring the robustness of SP11-induced transcriptional changes (Figure 3B). Trajectory heatmap analysis demonstrated coordinated regulation of biologically relevant genes along pseudotime (Figure 3B). In control cells, these genes showed progressive upregulation along the trajectory, while SP11-treated cells displayed suppressed or delayed expression dynamics. This shift may suggest that SP11 interferes with normal proliferative and stemness-related trajectories and redirects cells toward altered, stress-adaptive states. Functional enrichment analysis showed enrichment of pathways associated with cellular signaling, immune regulation, metabolism, and stress response (Figure 3C). Pathways related to transcriptional regulation, cell communication, and adaptive responses also appeared to be represented among the differentially expressed genes. These observations suggest that SP11 treatment may be associated with broader transcriptional alterations and potential changes in cellular states.

### 2.6. Integrated Transcriptomic Analysis Reveals Treatment-Specific Gene Expression Patterns and Pathway Enrichment

**Figure 4:**
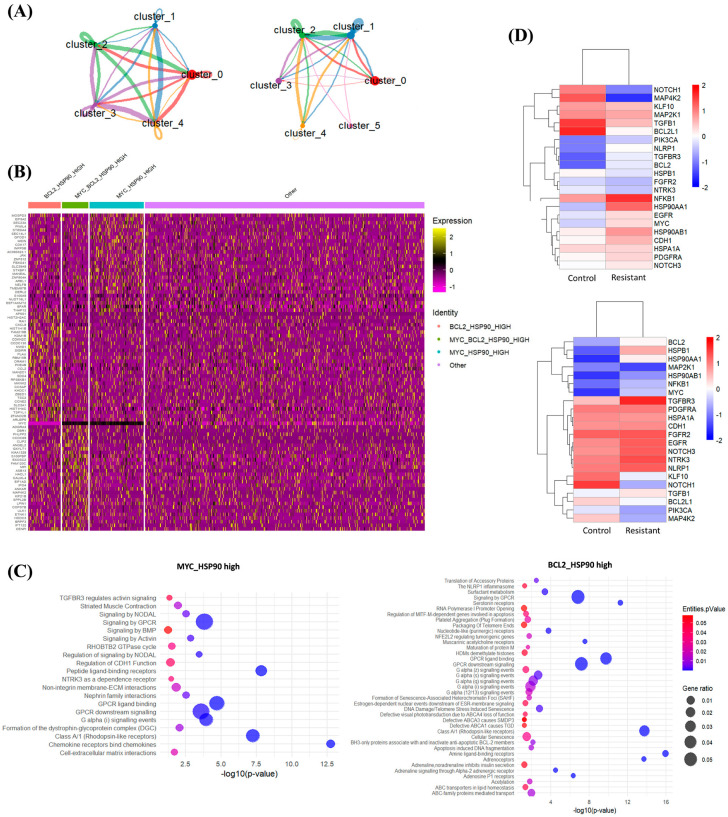
Single-cell analysis of SP11-treated MOLT4 cells highlights distinct survival pathways in *BCL2_HSP90* high and *MYC_HSP90* high subsets. (**A**) Cell–cell communication networks inferred from control (**left**) SP11-treated (**right**) clusters using CellChat. Circle nodes represent individual clusters, and the width of edges denotes the strength of interaction between clusters. (**B**) Heatmap of marker gene expression across cell fractions enriched for *BCL2* high/*HSP90* high, *BCL2* high/*MYC* high/*HSP90* high, and *MYC* high/*HSP90* high subsets compared to other populations. (**C**) Pathway enrichment analysis for *MYC* high/*HSP90* high (**left**) and *BCL2* high/*HSP90* high (**right**) cells. Bubble plots display significantly enriched pathways, with dot size representing the gene ratio, defined as the proportion of genes from the input gene set that are associated with a given pathway. Larger dots indicate higher gene ratios. and color indicating the adjusted *p*-value. MYC high cells showed dependency on growth factor and adhesion pathways (e.g., *TGFBR3*, *BMP*, *CDH1*, *NTRK3*), while *BCL2* high cells were enriched in apoptotic regulation, telomerase maintenance, and genome stability pathways. (**D**) Heatmap showing genes upregulated in SP11-resistant MOLT4 cells, associated with the aforementioned pathways, and highlighting key molecular signatures of resistance.

**Figure 5:**
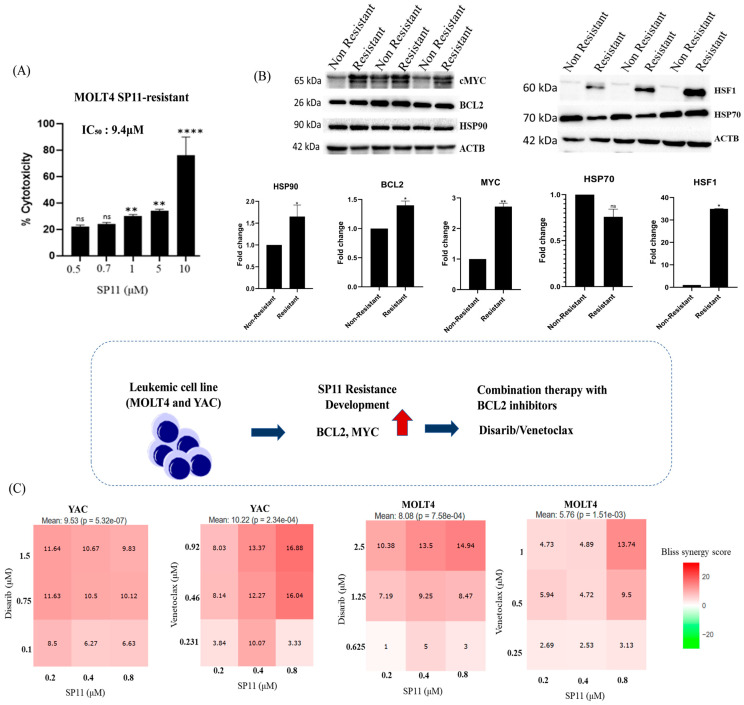
(**A**) Cytotoxicity analysis shows that SP11-resistant MOLT4 cells exhibit an IC_50_ of 9.4 µM (non-resistant IC_50_ is 0.8 µM), indicating markedly increased drug resistance. The x-axis represents SP11 concentration (µM), and the y-axis represents percentage cytotoxicity. Non- resistant represents the parental cell line. (**B**) Western blot analysis of *HSP90*, *MYC*, *BCL2*, *HSP70*, and HSF1 protein expression in SP11-resistant MOLT4 cells. The accompanying bar graph shows densitometric quantification of the blot, with expression levels normalized to *ACTB* and then to the basal expression level of parental non-resistant cells. Statistical significance was determined using an unpaired two-tailed Student’s *t*-test. *p* values: <0.05 = *, <0.01 = **, ≤0.0001 = ****, ns = non-significant. (**C**) BLISS synergy scores for *YAC* and *MOLT4* cells treated with a combination of SP11, Disarib, and Venetoclax at the indicated concentrations. The bliss synergy score is calculated using the SynergyFinder (v3) software.

To further explore transcriptional programs associated with resistance, we conducted integrated analyses of control and resistant populations. Cell–cell communication analysis showed significant rewiring of intercluster signaling networks in resistant cells, with changes in interaction strength and network structure compared to controls (Figure 4A). This indicates remodeling of intercellular communication under therapeutic pressure. Consistent with this, single-cell gene expression heatmaps revealed the emergence of distinct transcriptional subpopulations within resistant cells, including HSP90_BCL2 high, HSP90_MYC high, and HSP90_MYC_BCL2 high subsets (Figure 4B). These subpopulations displayed coordinated expression of genes related to survival, stress response, and resistance. Functional enrichment analysis indicated that MYC-high cells were enriched in pathways involved in growth factor signaling and cell adhesion (e.g., *TGFBR3*, *BMP*, *CDH1*, *NTRK3*), while *BCL2*-high cells were enriched in pathways governing apoptosis, telomerase maintenance, and genome stability (Figure 4C). These findings point to distinct, gene-specific survival mechanisms within resistant subpopulations. To validate these results, we developed an SP11-resistant MOLT4 cell line and performed bulk RNA sequencing. This revealed upregulation of *MYC*, *BCL2*, *MCL1*, and *NOTCH* pathway components (Figure 4D), aligning with the single-cell data. At the protein level, SP11-resistant MOLT4 cells showed increased expression of BCL2, MYC, HSP90, and HSF1, while HSP70 levels were decreased (Figure 5A). This suggests that resistant cells sustain oncogenic signaling while possibly avoiding excessive proteotoxic stress. Although these findings are consistent with adaptive resistance mechanisms, the possibility of clonal enrichment during prolonged drug exposure cannot be completely excluded. Given the crucial roles of MYC and BCL2 in resistance, we investigated combination therapeutic strategies. Treatment with BCL2 inhibitors (Venetoclax and Disarib) combined with SP11 produced strong synergistic effects in YAC and MOLT4, as reflected by high Bliss synergy scores (Figure 5C). We validated combination therapy using YAC cells, a lymphoma model characterized by high BCL2 expression, further supporting the therapeutic relevance of targeting BCL2 in combination with SP11.

### 2.7. Flow Cytometric, Cytokine, and Functional Analyses Validate Resistance-Associated Phenotypes

**Figure 6:**
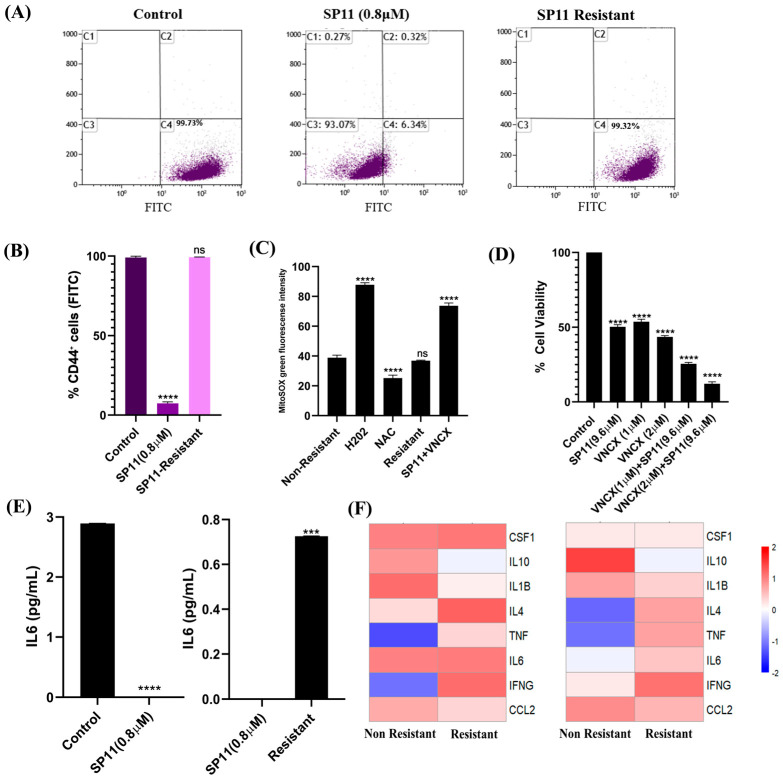
(**A**) Representative flow cytometry dot plots illustrating CD44 (FITC) surface expression in Control, SP11-treated (0.8 µM, 48 h), and SP11-resistant cells. Quadrant gating reveals shifts in cellular distribution across the experimental groups. (**B**) Quantitative analysis of the percentage of CD44-positive cells obtained from flow cytometry data. SP11 treatment markedly reduces the CD44-positive subpopulation, whereas SP11-resistant cells display elevated CD44 expression, reaching levels comparable to those observed in the control group. (**C**) Assessment of mitochondrial superoxide production using MitoSOX fluorescence intensity. Basal ROS levels in non-resistant cells were compared with a positive control (H_2_O_2_) and an antioxidant-treated negative control (NAC). SP11-resistant cells exhibited comparatively low basal ROS levels, which were markedly increased following 4 h co-treatment with Venetoclax (VNCX:2 µM) and SP11 (9.6 µM). (**D**) Cell viability analysis evaluating the cytotoxic effects of single-agent and combination treatments in SP11 resistant cells. Resistant cells were treated with SP11 (9.6 µM), VNCX (1 µM or 2 µM), or their combination. Combined SP11 and VNCX treatment produced enhanced cytotoxicity, resulting in significantly lower cell viability relative to individual treatments. (**E**) ELISA-based quantification of Interleukin-6 (IL6) secretion (pg/mL). The left panel shows a substantial reduction in IL6 secretion following acute SP11 treatment (0.8 µM) for 48 h in sensitive cells, whereas the right panel demonstrates significant re-elevation of IL6 secretion in SP11-resistant cells compared with the acute treatment condition. (**F**) Heatmaps showing differential expression profiles of inflammatory and immune-regulatory cytokines, including CSF1, IL10, IL1B, IL4, TNF, IL6, IFNG, and CCL2, between non-resistant and resistant cell populations across biological replicates. *p* values: <0.001 = ***, ≤0.0001 = ****, ns = non-significant.

Flow cytometric analysis demonstrated that SP11 treatment (0.8 µM) significantly reduced the proportion of CD44-positive cells compared with vehicle-treated controls (Figure 6A,B). In contrast, SP11-resistant cells maintained elevated CD44 expression, comparable to that of the control group (Figure 6A,B), indicating restoration of stem-like characteristics during resistance development. Furthermore, SP11 treatment at the IC50 for 48 h markedly suppressed IL-6 secretion, reducing cytokine levels to nearly undetectable levels relative to untreated controls (Figure 6E, left panel). However, resistant cells bypassed this suppressive effect and exhibited increased IL-6 secretion compared with acutely treated cells (Figure 6E, right panel). This restoration of cytokine signaling was accompanied by broader inflammatory transcriptomic remodeling, including altered expression of macrophage-associated and immune-regulatory mediators such as CSF1, IL10, IL1B, IL4, TNF, IFNG, and CCL2 between resistant and non-resistant populations (Figure 6F).

Given the persistence of stemness-associated and pro-survival signaling in resistant cells, strategies to overcome resistance were explored by targeting mitochondrial apoptotic pathways using the BCL-2 inhibitor Venetoclax (VNCX). Cell viability was first evaluated following single-agent and combination treatments. Resistant cells displayed limited sensitivity to SP11 (9.6 µM) or Venetoclax (1 µM or 2 µM) alone. In contrast, combination treatment produced a strong, dose-dependent reduction in viability (Figure 6D). To further investigate the underlying mechanism, mitochondrial reactive oxygen species levels were assessed using MitoSOX fluorescence intensity. Resistant cells exhibited relatively low basal ROS levels, comparable to those of non-resistant cells (Figure 6C). However, combined treatment with SP11 and Venetoclax (SP11 + VNCX) induced a marked increase in mitochondrial ROS accumulation, approaching levels observed in the hydrogen peroxide (H_2_O_2_) positive control (Figure 6C). These findings suggest that the enhanced cytotoxicity observed following combination treatment is associated with excessive mitochondrial oxidative stress and apoptotic vulnerability.

### 2.8. SP11 Treatment Remodels the Immune Landscape in the In Vivo DLA Tumor Microenvironment

**Figure 7:**
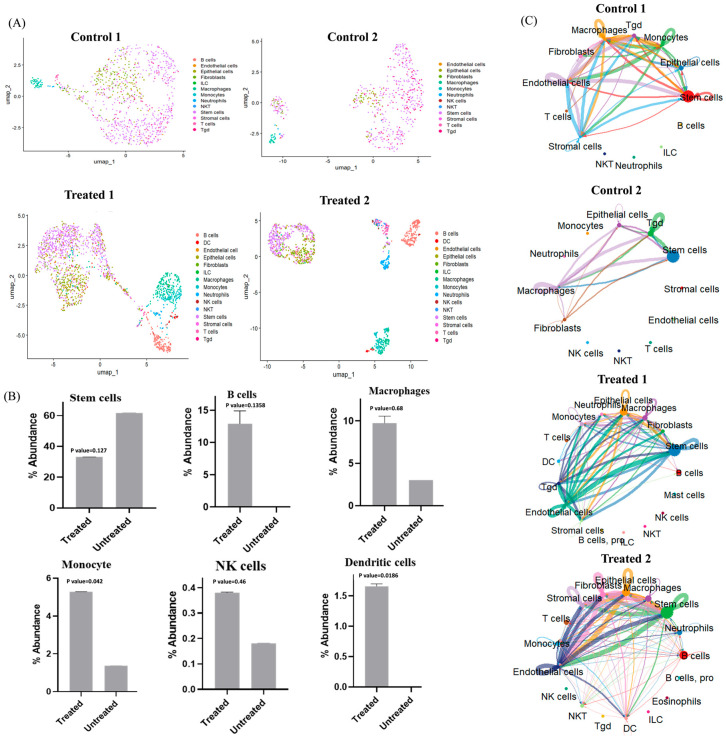
Single-cell landscape and cell–cell interaction analysis of control and SP11-treated DLA samples. (**A**) UMAP showing cellular heterogeneity in Control 1, Control 2, Treated 1, and Treated 2 samples. Each point represents an individual cell colored by annotated cell type, including B cells, T cells, NK/NKT cells, macrophages, monocytes, neutrophils, dendritic cells, fibroblasts, endothelial cells, epithelial cells, stromal cells, and stem cells. Clustering patterns illustrate treatment-associated shifts in cellular composition within the tumor microenvironment. (**B**) Quantitative comparison of selected immune and stem cell populations between treated and untreated conditions (*n* = 2 per condition), expressed as percentage abundance with corresponding statistical significance (Wilcoxon rank-sum test). Changes highlight alterations in stem cells (*p* value: 0.127), B cells (*p* value: 0.1358), macrophages (*p* value: 0.68), monocytes (*p* value: 0.042), NK cells (*p* value: 0.46), and dendritic cells (*p* value: 0.0186) following SP11 treatment. (**C**) Cell–cell communication networks inferred for Control 1, Control 2, Treated 1, and Treated 2 samples. Nodes represent distinct cell types, and edges indicate predicted ligand–receptor interactions, with edge thickness corresponding to interaction strength colors indicate the source cell type from which the interaction originates Network remodeling across conditions demonstrates treatment-induced changes in intercellular communication, particularly involving stem cells and immune populations.

To evaluate how SP11 affects the tumor microenvironment, single-cell transcriptomic profiling was performed on DLA tumor samples. UMAP visualization showed distinct differences in cellular organization between control and SP11-treated samples, indicating treatment-induced transcriptional modifications (Figure 7A). Clustering analysis identified several cellular subpopulations, with SP11-treated tumors displaying significant changes in cluster composition compared to controls. Quantitative analysis verified a decrease in dominant tumor-associated populations and an increase in specific immune cell populations following treatment. In control samples, tumor stem cells constituted the predominant population, with minimal immune cell infiltration. Conversely, SP11-treated samples showed a notable rise in immune cells, including B cells, monocytes, NK cells, dendritic cells, and macrophages, along with a significant reduction in stem cell populations (Figure 7A,B). This observation may reflect enhanced immune recruitment and a decrease in tumor-propagating cells after SP11 treatment. Cell–cell communication analysis also showed a comparatively increased number of signaling interactions between immune and tumor cells in treated samples (Figure 7C). This increased intercellular signaling could aid in tumor suppression and immune activation. However, these findings are based on computational transcriptomic analyses and require further experimental validation.

### 2.9. SP11 Modulates Specific Immune Subsets Associated with Tumor Progression and Immunity

γδ T cells in control samples showed increased expression of HSP90 and the anti-apoptotic marker BCL2, indicating a survival-adapted phenotype. γδ T cells demonstrate functional versatility in cancer, with some subsets exerting anti-tumor effects, while others promote tumor growth through immunosuppression and angiogenesis, especially IL-17–producing γδ T cells [28,29,30]. SP11-treated samples showed decreased γδ T cell levels, suggesting suppression of potentially pro-tumor immune subsets. Monocytes and macrophages, essential mediators of phagocytosis and cytokine-driven immune activation, increased after SP11 treatment (Figure 7A,B). Single-cell analysis revealed macrophage subsets co-expressing M1 markers (Il1b, Nos2) and M2 markers (Arg1, Mrc1, Il10), suggesting transitional polarization states rather than distinct M1 or M2 phenotypes (Supplementary Figure S2). Treated samples also showed relatively higher representation of neutrophils and dendritic cells, which may reflect altered inflammatory and antigen-presentation–associated responses.

We evaluated resistance markers such as MYC and BCL2 and observed an overall decrease after SP11 treatment. However, some cells retained high expression levels, suggesting a survival-related transcriptional program and potential development of resistance. Notably, B cells maintained BCL2 expression in treated samples, suggesting cell-specific regulation of anti-apoptotic pathways. Mechanistically, these results imply that SP11-mediated HSP90 inhibition could promote immune remodeling through interconnected pathways involving stress response, cytokine signaling, and adaptive feedback mechanisms.

### 2.10. Trajectory Analysis of Stem Cell Populations Reveals Residual Resistant States

**Figure 8:**
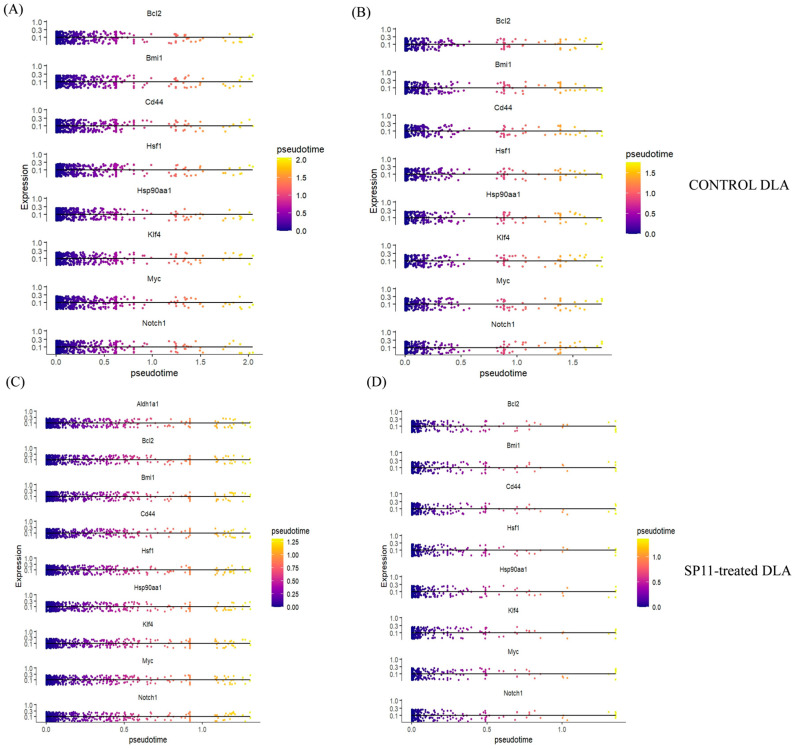
Trajectory-based pseudotime analysis of stemness and oncogenic gene expression in control and SP11-treated DLA cells. (**A**,**B**) Pseudotime expression patterns of *Bcl2*, *Bmi1*, *Cd44*, *Hsf1*, *Hsp90aa1*, *Klf4*, *Myc*, and Notch1 in the stem cell population of control DLA samples ((**A**): Control 1; (**B**): Control 2). (**C**,**D**) Dynamic expression profiles of the same genes within the stem cell population of SP11-treated DLA samples along the inferred pseudotime trajectory ((**C**): Treated 1; (**D**): Treated 2). Each dot represents an individual cell and is color-coded by pseudotime progression (purple-to-yellow gradient). The x-axis corresponds to pseudotime progression, whereas the y-axis represents normalized gene expression levels.

**Figure 9:**
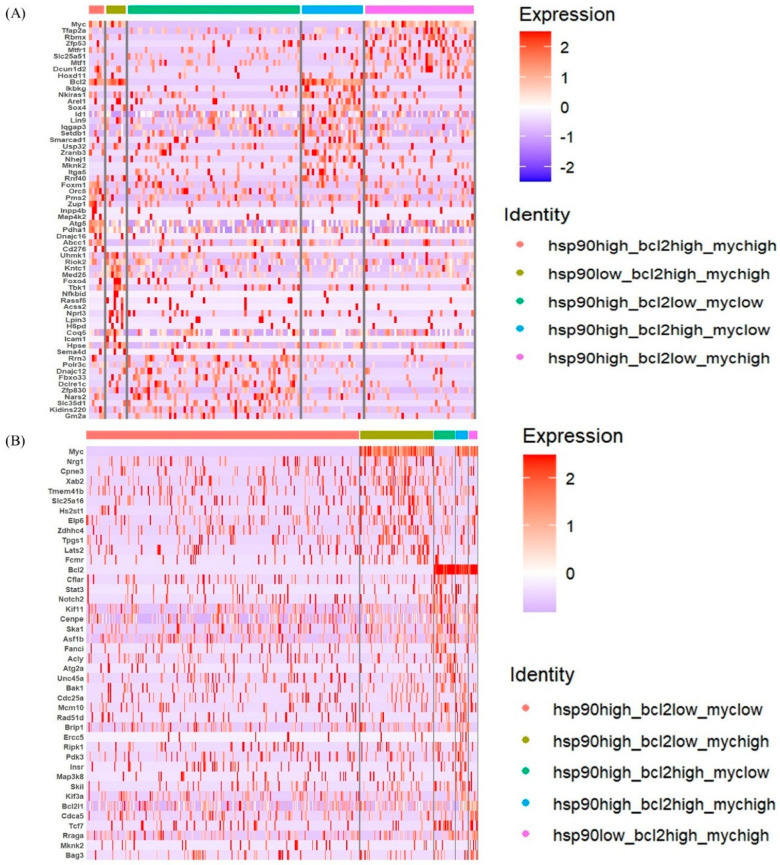
Heatmaps showing the expression of gene signatures across five cell populations in the SP11-treated DLA stem cell population. (**A**) corresponds to SP11-treated 1, and (**B**) corresponds to SP11-treated 2. Columns represent individual cells grouped by cluster identity based on *Hsp90*, *Bcl2*, and *Myc* expression profiles, while rows indicate selected marker genes. Red denotes high expression, and dark blue/purple represents low expression. Distinct clusters exhibit differential enrichment of specific marker genes, highlighting subtype-specific transcriptional programs and treatment-associated shifts in stem cell population dynamics.

**Figure 10:**
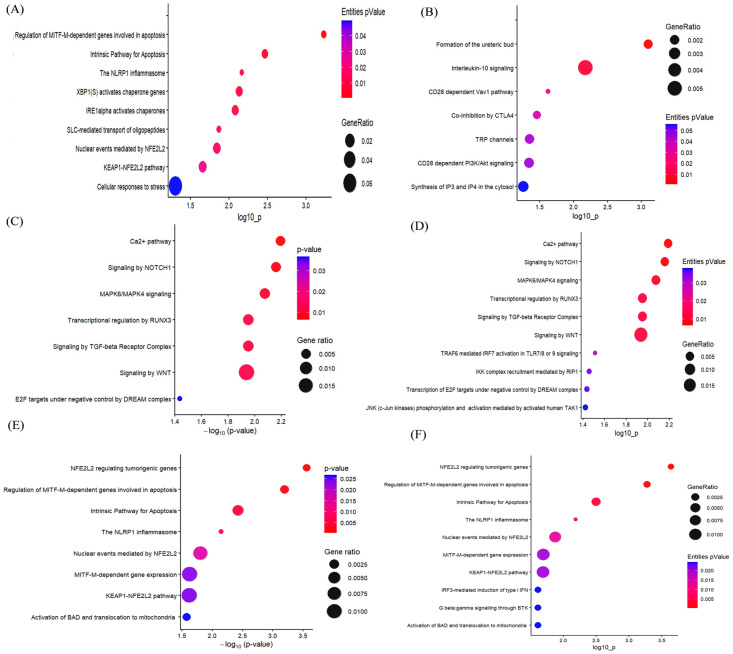
Pathway enrichment analysis in the stem cell population of SP11-treated cells. Dot plots showing significantly enriched pathways in SP11-treated sample 1 (panels (**A**,**C**,**E**)) and SP11-treated sample 2 (panels (**B**,**D**,**F**)). Panels A and B represent the Bcl2/Myc/Hsp90aa1 high cluster, (panels (**C**,**D**)) represent the Myc/Hsp90aa1 high cluster, and (panels (**E**,**F**)) represent the Bcl2/Hsp90aa1 high cluster. The x-axis represents log10(*p*-value), indicating the statistical significance of pathway enrichment. Dot size reflects gene ratio, defined as the proportion of genes from the input gene set that are associated with a given pathway. Larger dots indicate higher gene ratios and color intensity corresponds to adjusted *p*-value.

Based on SingleR-derived annotations, stem cell subsets from control and SP11-treated DLA samples were analyzed using trajectory inference. SP11 treatment showed a significant reduction in key stemness and survival regulators, including BCL2, MYC, HSP90AA1, HSF1, ALDH1A1, BMI1, CD44, KLF4, and NOTCH1 (Figure 8), indicating disruption of stem cell maintenance programs. However, a distinct subset of treated cells maintained high expression of these markers, suggesting the persistence of residual stem-like populations capable of adaptive survival. To better characterize these populations, treated cells were stratified based on Myc, Bcl2, and Hsp90aa1 expression signatures (Figure 9). Each subgroup exhibited unique transcriptional profiles, highlighting heterogeneity in adaptive responses. Myc-high cells mainly activated proliferative and metabolic pathways, including WNT, NOTCH, TGF-β, and MAPK signaling (Figure 10A–D). These cells also showed increased expression of genes involved in DNA damage tolerance (Rbmx, Ercc6l2) and metabolic adaptation (Slc27a1, Mtfr1), indicating a possible increase in proliferative and metabolic capacity. In contrast, Bcl2-high cells were enriched in pathways associated with resistance to apoptosis and immune modulation, including intrinsic apoptotic regulation, NRF2-mediated oxidative stress responses, inflammasome activation, and STING-dependent signaling (Figure 10 A,B,E,F). This phenotype was supported by sustained Bcl2 expression, along with genome stability genes (Prkdc, Dclre1a) and immune-related genes (Mcemp1, Stx11), suggesting improved survival under cytotoxic stress. The Bcl2/Myc/Hsp90aa1-high population could be a particularly aggressive and therapy-resistant state, integrating proliferative, survival, and immune evasion mechanisms.

This group showed activation of PI3K/AKT signaling, immune co-inhibitory pathways, and calcium signaling, as well as increased expression of genes involved in cytoskeletal remodeling (Fgd4, Prickle4), which might support invasion and cellular plasticity. Furthermore, this population exhibited elevated levels of stress-tolerance regulators (Bnipl, Hsf2bp), immune-regulatory molecules (Cd86, Itpkc), and the stemness-related transcription factor Six2, which may reflect adaptive, self-renewal, and stress-responsive transcriptional features (Figure 9A,B).

## 3. Discussion

Our study suggests that the novel HSP90 inhibitor SP11 may exert anti-leukemic effects through both inhibition of tumor-intrinsic oncogenic signaling and modulation of the tumor microenvironment in T-ALL and lymphoma models, respectively. Using single-cell transcriptomic profiling, we observed that SP11 reduces key oncogenic drivers, including MYC, BCL2, and regulators of stemness, and enhances immune activity and cytokine levels within the tumor environment. These findings suggest that SP11 acts not only as a cell-killing agent but also as a modulator of tumor–immune interactions.

HSP90 plays a vital role in maintaining proteostasis by stabilizing many oncogenic client proteins that support cancer growth and resistance to therapy [8]. Despite strong preclinical evidence, many HSP90 inhibitors, such as AUY922, BIIB021, and geldanamycin derivatives, have shown limited success in clinical trials due to toxicity and the development of resistance [31,32]. In this context, SP11 exhibits potent anti-leukemic activity at very low concentrations and is effective in both cell culture and animal models [10], supporting its potential as a next-generation HSP90 inhibitor.

A key finding is the consistent reduction in MYC and BCL2 following SP11 treatment. These proteins are central to oncogenic signaling and are associated with aggressive disease and resistance in lymphoid cancers [11,12,13]. Their coordinated suppression indicates that SP11 effectively interrupts essential survival pathways in T-ALL. However, single-cell analysis reveals significant heterogeneity in how cells respond within tumors. Despite overall suppression of oncogenic programs, some cells maintain or reactivate MYC and BCL2, indicating early adaptive resistance.

This resistant group aligns with the concept of drug-tolerant/adaptive cells, which develop under therapy through changes in gene expression. Trajectory analysis further supported these observations, showing that SP11-treated cells may shift from proliferative and stem-like states toward stress-response pathways. These findings highlight the potential role of cellular plasticity in influencing therapy response and the importance of single-cell analysis in identifying resistant cell states.

The results are reinforced by the development of an SP11-resistant MOLT4 model, which displays increased levels of MYC, BCL2, and related survival pathways at both the gene and protein levels. This concordance across data types indicates that resistance to SP11 results from coordinated adaptive responses. Mechanistically, these findings are consistent with previous studies showing that inhibiting HSP90 can activate compensatory pathways such as HSF1, NF-κB, and PI3K/AKT, which help cells survive under stress [14,33,34,35].

Beyond effects within tumor cells, SP11 could cause significant changes in the tumor immune microenvironment in the DLA model. Treated tumors showed increased infiltration of immune cells like monocytes, macrophages, dendritic cells, NK cells, and B cells, along with enhanced cell–cell communication networks. These results could suggest that SP11 fosters a more active immune environment. Notably, increased antigen-presenting cells and immune signaling pathways align with earlier studies indicating that HSP90 inhibition may boost anti-tumor immunity by elevating interferon responses and improving antigen presentation [36,37].

At the same time, the immune landscape appeared dynamic and heterogeneous. Macrophages expressing both M1 and M2 markers suggest transitional states, reflecting immune shifts under therapy. Additionally, the persistence of BCL2-high immune cells and a reduction in γδ T cells could indicate selective changes within immune compartments that may influence tumor suppression and immune evasion.

Importantly, identifying MYC/BCL2-high subpopulations provided a strong rationale for combination therapies. Validation studies show that combining BCL2 inhibitors like Venetoclax or Disarib with SP11 significantly increases sensitivity, offering a promising strategy to overcome resistance. Since BCL2 inhibitors are already approved for clinical use, these findings have immediate translational relevance.

In summary, this work provides a detailed single-cell view of how T-ALL responds to and develops resistance against HSP90 inhibitors. By linking tumor-intrinsic signaling, adaptive resistance, and immune modulation, our findings offer key insights to guide the development of new therapies to improve outcomes in aggressive blood cancers. There are several limitations to this study: the cell line studies do not fully reflect the complexity of human T-ALL, and the DLA lymphoma model does not completely recapitulate human T-ALL biology, although it serves as a useful immunocompetent hematological tumor model for investigating SP11-associated transcriptional and tumor microenvironmental responses in vivo. Further studies using PDX or genetically engineered mouse models would strengthen the translational value. More comprehensive cytokine profiling and immune functional assays would further strengthen the characterization of immune remodeling. Combination therapy and validation in treatment-resistant patient samples are required for validating the significance of this study.

## 4. Methodology

### 4.1. Single-Cell RNA Sequencing

- In vitro model (MOLT4): MOLT4 cell (ATCC, USA) were cultured in RPMI-1640 supplemented with 10% fetal bovine serum and 1% penicillin-streptomycin (Gibco, South American) at 37 °C in a 5% CO_2_ incubator. Cells were treated with SP11 at 0.8 µM (IC50) for 48 h. After treatment, cells were harvested for single-cell RNA sequencing (scRNA-seq). The experiment was performed in replicates.
- In vivo model (DLA): The study received approval from the Committee for the Control and Supervision of Experiments on Animals (CPCSEA, Government of India, Animal Welfare Division, Reg. No. 1994/GO/ReBi/S/17/CPCSEA). All procedures were conducted in accordance with institutional and national CPCSEA guidelines. To induce a tumor, DLA cells (1 × 10^6^ cells per animal) were injected intraperitoneally in Swiss albino mice. Once tumors were established, mice were treated with SP11 at a dose of 30 mg/kg body weight, administered orally for 17 days. After treatment, mice were sacrificed, and tumors were collected for scRNA-seq analysis. Two independent sets of control and SP11-treated samples were used for the experiment.
- Library preparation and sequencing: Single-cell suspensions with high viability (>85%) were prepared, filtered, and loaded onto the 10× Genomics Chromium platform (10× Genomics, Pleasanton, CA, USA) for GEM generation. Cells were partitioned into nanoliter-scale Gel Bead-in-Emulsions (GEMs) using the Chromium Controller, ensuring each droplet ideally contained a single cell and a barcoded gel bead. During cell lysis within GEMs, mRNA transcripts were captured by bead-bound oligonucleotides containing cell-specific barcodes and unique molecular identifiers (UMIs), followed by reverse transcription to produce barcoded cDNA. The emulsion was then broken, and cDNA was purified using magnetic beads and amplified by PCR. Sequencing libraries were prepared by fragmentation, end repair, A-tailing, adaptor ligation, and index PCR, and quality was assessed using the Agilent Bioanalyzer (Agilent Technologies, Santa Clara, CA, USA). Final libraries were sequenced on an Illumina platform (Illumina Inc., San Diego, CA, USA) with paired-end reads to capture cell barcodes, UMIs, and transcript sequences.
- Downstream analysis: After sequencing, raw data were processed using the Cell Ranger pipeline (10× Genomics) for demultiplexing, alignment to the reference genome (hg38), and generation of gene-barcode matrices. The Seurat package (v5.3.0) was used for downstream analysis. Data normalization and scaling were performed using the standard Seurat workflow, following the guidelines provided by the Satija Lab https://satijalab.org/seurat/ (accessed on 3 April 2025). Differential expression was assessed using the Wilcoxon rank-sum test with adjusted *p* < 0.05 and logFC > 0.25 or 0.5.

### 4.2. Cell Type Identification

Cell type annotation was performed using the SingleR package (v2.10.0), which compares single-cell transcriptomes to reference datasets from the celldex package (v1.18.0) and assigns cell types based on expression similarity. Cell cycle phases were assigned using Seurat’s CellCycleScoring() function, based on predefined S-phase and G2M-phase marker genes.

### 4.3. Trajectory Analysis

To investigate cellular differentiation pathways, trajectory analysis was performed using Monocle 3 (v1.4.25). The Seurat-scaled and normalized data were converted into a CellDataSet object compatible with Monocle 3. Dimensionality reduction was achieved using Uniform Manifold Approximation and Projection (UMAP). Pseudotime analysis was conducted to order cells along inferred trajectories, providing insights into dynamic biological processes.

### 4.4. Development of SP11-Resistant MOLT4

SP11-resistant MOLT4 cells were generated by continuous exposure to increasing concentrations of SP11 (starting from the parental IC_50_ of 0.8 μM) and stepwise dose increases as cells adapted. Cultures were maintained in drug-containing medium for several weeks until stable growth was achieved at high concentrations. Resistance was confirmed by MTT assay, which showed a significant increase in IC_50_ (9.6 μM) compared to parental cells, and by Western blot analysis, which revealed elevated HSP90 expression in resistant cells relative to controls. Western blot band intensities were quantified using ImageJ software v1.53e (National Institutes of Health, Bethesda, MD, USA) and normalized to ACTB as a loading control. Bulk RNA-seq libraries were prepared from resistant and parental cells, and differential expression was analyzed using DESeq2 (v1.48.1).

### 4.5. Lactate Dehydrogenase Assay

For the LDH cytotoxicity assay, cells were seeded in 96-well plates at a density of 1 × 10^4^ cells per well and treated with varying drug concentrations in culture medium for 48 h. After incubation, the plates were centrifuged, and the supernatants were transferred to fresh 96-well plates. The LDH assay reagents (MP Biomeicals, Irvine, CA, USA) was then added, and the mixture was incubated for 15 min at 37 °C to allow the enzymatic reaction to proceed. Absorbance was measured at 490 nm using a microplate reader. Drug-induced inhibition percentages were calculated relative to untreated control wells, and IC_50_ values were determined from dose–response curves using GraphPad Prism 8 (GraphPad Software, San Diego, CA, USA).

### 4.6. Western Blot Analysis

Total protein was extracted from drug-treated cell pellets using RIPA buffer. Proteins were separated by SDS-PAGE on 12% polyacrylamide gels (acrylamide:bis-acrylamide, 29:1) based on their molecular weight. The resolved proteins were transferred onto PVDF membranes (Bio-Rad Laboratories, Hercules, CA, USA) using a wet transfer system at 100 V for 2 h (Bio-Rad Laboratories, Hercules, CA, USA). Following transfer, membranes were blocked with 5% skim milk in PBST (1× PBS containing 0.1% Tween-20) for 1 h at room temperature. Membranes were then incubated with specific primary antibodies overnight at 4 °C. After washing three times with PBST (15 min each), membranes were incubated with the appropriate secondary antibodies (1:1000 dilution) for 1 h at room temperature. After additional washing steps, protein bands were visualized using the Bio-Rad ECL (Bio-Rad Laboratories, Hercules, CA, USA) chemiluminescence detection system and imaged with a gel documentation system.

### 4.7. Flow Cytometric Analysis of CD44 Expression

MOLT4 cells were seeded in 6-well plates and treated with SP11 (0.8 µM) for 48 h. Following treatment, cells were collected by centrifugation and washed twice with cold PBS. Approximately 1 × 10^6^ cells were resuspended in PBS containing 1% BSA and incubated with CD44 Monoclonal Antibody (IM7, BioLegend, San Diego, CA, USA), FITC for 30 min at 4 °C in the dark. After incubation, cells were washed twice with PBS and resuspended in 300–500 µL PBS for acquisition. Flow cytometric analysis was performed using a Beckman Coulter Gallios Flow Cytometer (Beckman Coulter, Brea, CA, USA), and FITC fluorescence was detected in the FL1 channel. A minimum of 10,000 events were acquired per sample. Data were analyzed using Kaluza (v2.3, Beckman Coulter, Brea, CA, USA), and CD44 expression was quantified relative to vehicle-treated control cells. All experiments were performed in triplicate.

### 4.8. Measurement of IL-6 Secretion by ELISA

IL-6 levels were measured using the Human IL-6 (Interleukin 6) ELISA Kit (E-EL-H6156, Elabscience Biotechnology Inc., Houston, TX, USA) according to the manufacturer’s instructions. Briefly, MOLT4 cells were treated with SP11 (0.8 µM) for 48 h, and culture supernatants were collected for analysis. Subsequently, 100 µL of standards or samples were added to ELISA plate wells and incubated for 90 min at 37 °C. After removal of the liquid, 100 µL of biotinylated detection antibody working solution (1:99 dilution) was added and incubated for 60 min at 37 °C. The plate was washed three times, then 100 µL of HRP conjugate working solution (1:99 dilution) was added and incubated for 30 min at 37 °C. After washing five times, 90 µL of TMB substrate was added, and the mixture was incubated for 15 min at 37 °C. The reaction was stopped with 50 µL stop solution, and absorbance was measured immediately at 450 nm.

### 4.9. Mitochondrial ROS Measurement Using MitoSOX™ Green

Mitochondrial superoxide generation was assessed using the MitoSOX™ Green Mitochondrial Superoxide (Invitrogen™, Thermo Fisher Scientific, Waltham, MA, USA) Indicator according to the manufacturer’s protocol. Briefly, cells were seeded in 96-well plates and treated with venetoclax (2μM) and SP11 (9.6μM) for 4 h. Hydrogen peroxide (H_2_O_2_; 500 µM for 30 min) was used as a positive control for ROS induction, whereas N-acetyl-L-cysteine (NAC; 10 mM for 1 h) served as a negative control. Following treatment, cells were incubated with 1 µM MitoSOX™ Green working solution for 30 min at 37 °C in the dark, washed with PBS, and fluorescence intensity was measured using a microplate reader at excitation/emission wavelengths of 488/510 nm. Fluorescence values were normalized to vehicle-treated control wells.

### 4.10. Statistical Analyses

Statistical analyses were conducted using RStudio (version 4.4.2) and GraphPad Prism 8.4.2. Comparisons between treatment groups and their respective controls were performed using Student’s *t*-test or one-way analysis of variance (ANOVA), followed by Tukey’s multiple-comparison post hoc test when applicable. Results are presented as mean ± standard deviation (SD). A *p*-value ≤ 0.05 was considered statistically significant and represented as * (*p* ≤ 0.05), ** (*p* ≤ 0.01), *** (*p* ≤ 0.001), and **** (*p* ≤ 0.0001), while ns indicates non-significant differences.

## 5. Conclusions

HSP90 inhibition by SP11 was associated with suppression of oncogenic signaling and stemness-related pathways, as well as remodeling of the tumor immune microenvironment. SP11 treatment was associated with altered immune cell representation, immune-related transcriptional signatures, immune activation, and enhanced immune cell infiltration, suggesting effects on both tumor cells and the surrounding immune milieu. Nevertheless, the persistence of MYC/BCL2-high tumor cells and BCL2-high, M2-like macrophage populations indicates the potential emergence of adaptive resistance mechanisms. Collectively, these findings underscore the complex and context-dependent role of HSP90 as a therapeutic target and highlight the importance of rational combination strategies. In particular, simultaneous targeting of survival pathways such as BCL2, together with modulation of the immune microenvironment, may be important for overcoming resistant cellular populations and improving the durability of anti-tumor responses.

## Data Availability

The datasets generated during this study are publicly available in the NCBI Gene Expression Omnibus (GEO) repository under accession numbers GSE328097 and GSE328525, which are linked to BioProject accession number PRJNA1453909.

## Supplementary Materials

The following supporting information can be downloaded at: https://www.mdpi.com/article/10.3390/ijms27125321/s1.
