# Supplementary material for: Single-Cell Transcriptomic Profiling Reveals Dual Antitumor and Adaptive Resistance Mechanisms of a Novel HSP90 Inhibitor, SP11, in T-Cell Acute Lymphoblastic Leukemic Cells and DLA Mouse Model
Source: Int J Mol Sci. 2026 Jun 12;27(12):5321. doi: 10.3390/ijms27125321 (PMC13299467; doi:10.3390/ijms27125321)
Supplement: Supplementary file 1 [file ijms-27-05321-s001.zip › ijms-4291730-supplementary.pdf]

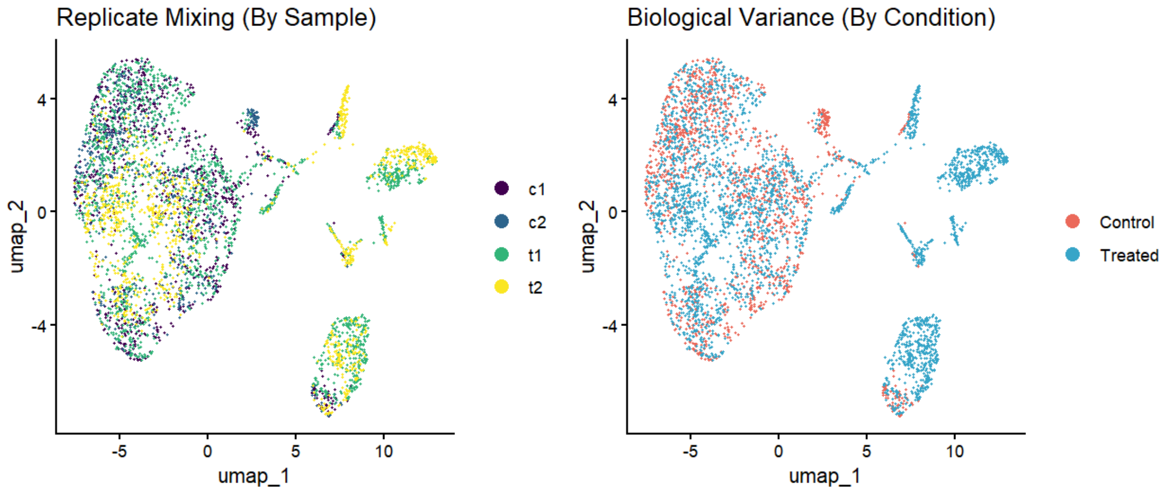

Figure 1: Exploratory assessment of batch effects and replicate mixing across scRNA-seq datasets.
Single-cell RNA-sequencing datasets from two control samples (c1 and c2) and two treated samples (t1 and t2) were merged and processed using a common analysis workflow, including normalization, variable feature selection, principal component analysis (PCA), and UMAP dimensionality reduction, without applying computational batch-correction or integration methods. UMAP visualization demonstrated substantial overlap and intermixing of cells across biological replicates within each condition, with clustering patterns primarily reflecting biological states rather than sample-specific technical variation, indicating minimal batch-driven effects.

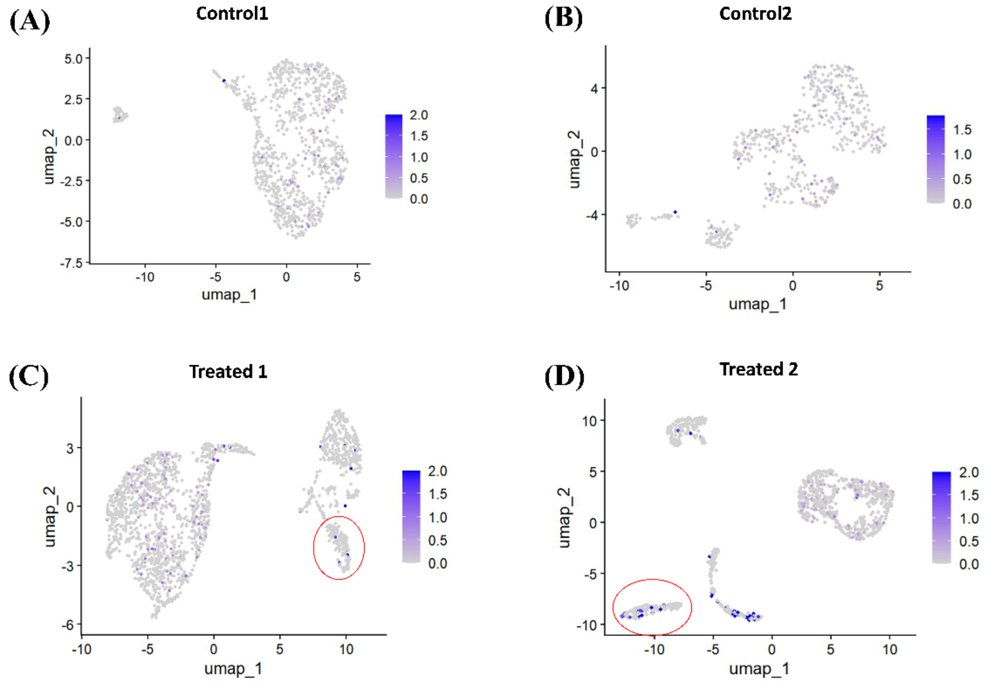

Figure 2: Single-cell transcriptomic profile of BCL2 *expression across control and SP11-treated DLA models. UMAP plots displaying scRNA-seq expression levels of the anti-apoptotic marker* BCL2 *across four experimental conditions: (A) Control 1, (B) Control 2, (C) Treated 1 (SP11), and (D) Treated 2 (SP11). Expression intensity is color-coded according to the gradient scale (grey indicates low/no expression; blue indicates high expression). Cells were annotated using SingleR. Red circles highlight a distinct B-cell population in the treated cohorts that retains* BCL2 *transcript levels post-treatment.*

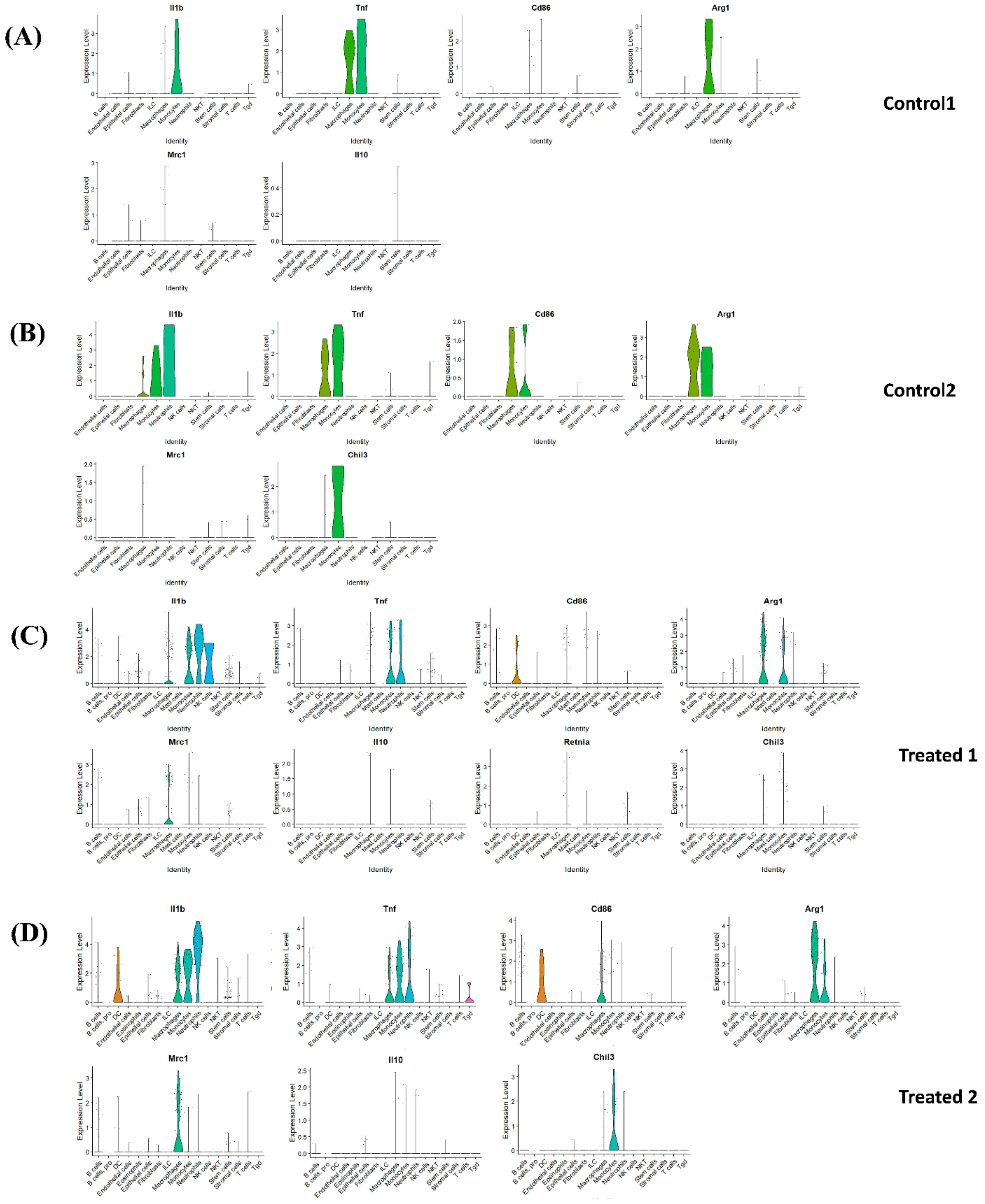

Figure 3: Expression profiles of macrophage polarization and activation markers across control and SP11-treated DLA conditions. Violin plots generated from single-cell RNA sequencing data illustrating the transcript expression levels of key macrophage phenotypic markers across four experimental setups: (A) Control 1, (B) Control 2, (C) Treated 1 (SP11), and (D) Treated 2 (SP11).

The paneled markers include canonical pro-inflammatory (M1-like) indicators-such as Il1b, Tnf, and Cd86- alongside anti-inflammatory, tissue-remodeling, or regulatory (M2-like) indicators including Arg1, Mrc1 ,Il10, Chil3, and Retnla.

Transcripts are plotted across annotated cellular identities along the x-axis, highlighting dynamic shifts in expression primarily localized within the myeloid compartments (macrophages and monocytes) between the vehicle controls and SP11-treated groups.
